# Seroprevalence and prognostic value of *Aspergillus*-specific IgG among non-neutropenic invasive pulmonary aspergillosis patients: a prospective multicenter study

**DOI:** 10.1186/s41479-024-00154-8

**Published:** 2024-11-05

**Authors:** Meng-Rui Lee, Hsu-Liang Chang, Yung-Hsuan Chen, Chia-Jung Liu, Li-Ta Keng, Hung-Ling Huang, Jann-Yuan Wang, Chau-Chyun Sheu, Inn-Wen Chong

**Affiliations:** 1 Department of Internal Medicine, National Taiwan University Hospital, Taipei, Taiwan; 2 Department of Internal Medicine, National Taiwan University Hospital Hsin-Chu Branch, Hsin-Chu, Taiwan; 3 Division of Pulmonary and Critical Care Medicine, Kaohsiung Medical University Hospital, Kaohsiung, Taiwan; 4 Department of Internal Medicine, Kaohsiung Medical University Hospital, Kaohsiung, Taiwan; 5 Department of Internal Medicine, Kaohsiung Municipal Ta-Tung Hospital, Kaohsiung, Taiwan; 6 Department of Internal Medicine, School of Medicine, Kaohsiung Medical University, Kaohsiung, Taiwan; 7 Graduate Institute of Medicine, College of Medicine, Kaohsiung Medical University, Kaohsiung, Taiwan; 8 Departments of Respiratory Therapy, Kaohsiung Medical University Hospital, Kaohsiung, Taiwan; 9 Center for Liquid Biopsy and Cohort Research, Kaohsiung Medical University, Kaohsiung, Taiwan; 10 Department of Biological Science and Technology, National Yang Ming Chiao Tung University, Hsinchu, Taiwan

**Keywords:** *Aspergillus fumigatus*, *Aspergillus* IgG, Invasive pulmonary aspergillosis, Diagnosis, Outcome

## Abstract

**Background:**

This study aimed to assess the diagnostic and prognostic value of *Aspergillus*-specific IgG (*Asp*-IgG) for invasive pulmonary aspergillosis (IPA) in non-neutropenic non-hematologic patients.

**Methods:**

Between November 2019 and February 2022, we recruited 40 non-neutropenic, non-hematologic IPA patients from Taiwan and measured serum *Asp*-IgG levels using Phadia, Thermofisher. A positive *Asp*-IgG test was defined as a level > 40 mgA/L. We evaluated the association between *Asp*-IgG levels and overall survival, as well 90-day mortality rate of IPA patients.

**Results:**

Of the 40 participants, 11 (27.5%) tested positive for *Asp*-IgG, while 16 (40%) had positive galactomannan antigen (optical density > 1). Higher *Asp*-IgG levels were associated with improved overall survival (HR: 0.22, 95% CI: 0.05–0.99, *p* = 0.035) in multivariable Cox regression. The overall 90-day mortality rate was 65% (26/40). We found that patients with low *Asp*-IgG levels (≤ 40 mgA/L) had a borderline higher 90-day mortality rate compared to patients with high *Asp*-IgG levels (OR: 3.15, 95% CI: 0.75–13.28, *p* = 0.118). Stratifying by serum galactomannan and *Aspergillus* IgG levels, patients with elevated serum GM and low *Asp*-IgG had the highest 90-day mortality (80%, 8/10), followed by patients with low serum GM and low *Asp*-IgG (68.4%, 13/19).

**Conclusions:**

*Asp*-IgG was positive in approximately one-fourth of non-neutropenic IPA patients. *Asp*-IgG may hold potential as a clinical prognostic factor for IPA. Further studies are required to validate this finding.

**Supplementary Information:**

The online version contains supplementary material available at 10.1186/s41479-024-00154-8.

## Introduction

Invasive pulmonary aspergillosis (IPA) is a serious fungal disease that has a high mortality rate ranging from 30 to 90% [1]. Delayed and even failure in initiating appropriate antifungal agents lead to increased mortality rate and adverse outcome [2]. While IPA was traditionally associated with immunocompromised hematologic disease patients, the recent influenza and COVID-19 pandemics have expanded its host population, leading to an increase in reported cases [3, 4]. Nevertheless, the current diagnostic tests for IPA are insufficient, as fungal cultures are often ineffective and obtaining pathology proof can be difficult [5]. Only 20% to 40% of Aspergillus culture tests are positive, and proof of pathology is often unavailable due to debilitated conditions of the patients [6]. In addition, prognostic biomarkers for disease outcomes are not readily available [7]. Non-culture-based methods for diagnosing IPA are now considered crucial for improving the accuracy of diagnosis [5]. Although the galactomannan (GM) test in serum or bronchoalveolar lavage (BAL) is now the central tool for diagnosing IPA, it is not without limitations. The serum GM test's sensitivity may be limited in non-neutropenic hosts, and the BAL GM test requires invasive procedures [8].

However, it is not clear whether *Aspergillus*-specific immunoglobulin G (*Asp*-IgG), which is commonly used to detect chronic pulmonary aspergillosis (CPA), can be used to diagnose the more invasive and early form of pulmonary aspergillosis [9]. In cases of neutropenic invasive pulmonary aspergillosis (IPA), the inability to produce antibodies in a neutropenic state has reduced the effectiveness of *Asp*-IgG as a diagnostic tool [10]. In non-neutropenic patients, *Asp*-IgG may have certain role in IPA diagnosis [11, 12] and immunoglobulin G is the major type of antibody for humoral immunity [13]. A study conducted in China found that *Asp*-IgG levels were higher in patients with CPA and non-neutropenic IPA compared to those with bacterial pneumonia or healthy participants [11]. Another study evaluating the performance of serum galactomannan (GM), (1–3)-β-D-glucan (BG), and *Asp*-IgG in diagnosing non-agranulocytic IPA found that integrating *Asp*-IgG into the final analysis improved overall sensitivity [12]. Moreover, there was a tendency for higher *Asp*-IgG levels in the group of patients who survived, although this was statistically insignificant (*p* = 0.268) [12]. These findings suggest that *Asp*-IgG could potentially be a useful diagnostic and prognostic tool for non-neutropenic IPA.

The objective of this study was, therefore, to examine the practical use of serum *Asp*-IgG in diagnosing IPA and predicting its outcome.

## Materials and methods

### Ethics statement

This study was prospectively conducted in accordance with ethical principles and standards. The Institutional Review Boards of all participating hospitals granted approval for the study (Approval numbers: 108–079-E and KMUHIRB-E(I)-202001118). Informed consent was obtained from all study participants prior to their inclusion in the study.

### Study participants and setting

**Fig. 1:**
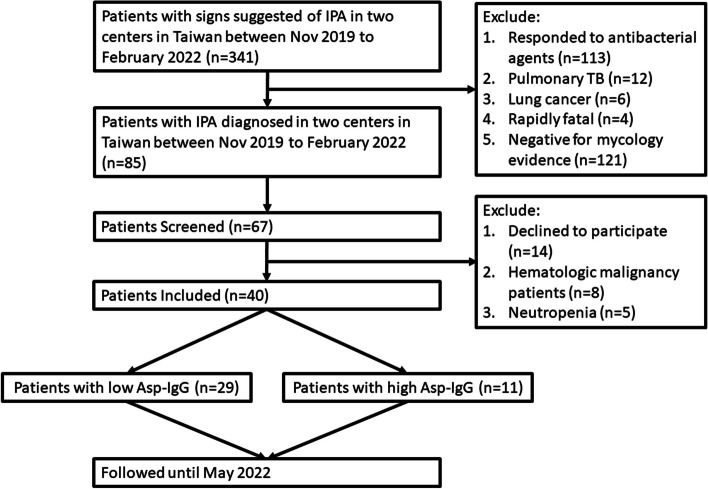
Flow-chart of patient recruitment

This prospective study was conducted at two hospitals located in northern Taiwan (National Taiwan University Hospital Hsin-Chu branch [NTUH-HC]) from November 2019 to February 2022, and southern Taiwan (Kaohsiung Medical University Hospital Kaohsiung Municipal Ta-Tung Hospital [KMUH-MT]) from July 2020 to February 2022. We screened patients who were clinically suspected for IPA and among them, we enrolled patients who were diagnosed with IPA thereafter. Follow-up of patients continued until May 2022. Figure 1 depicts the study flow-chart.

### Measurement of Serum Asp-IgG Level

The levels of *Asp*-IgG were measured using automated ImmunoCAP systems (Phadia 100; Thermo Fisher Scientific, Uppsala, Sweden) in this study [14]. *A. fumigatus*-specific IgG and *A. niger*-specific IgG were measured for each serum sample collected at the time of recruitment. For the purposes of this manuscript, the term *Aspergillus*-specific IgG referred specifically to *Aspergillus fumigatus*-specific IgG, unless otherwise stated.

### Definition and data collection

The diagnosis of invasive pulmonary aspergillosis (IPA) is confirmed through histo-pathology evidence as per the criteria of the European Organization for Research and Treatment of Cancer and the Mycoses Study Group Education and Research Consortium (EORTC/MSGERC) [15]. Probable diagnosis of IPA is made using the BM-AspICU criteria, which combine the EORTC/MSGERC and Asp-ICU criteria [15–17]. Briefly, the proven IPA patients required histological evidence or a positive *Aspergillus* culture in a sterile site [15]. The diagnostic criteria of probable IPA included the following: (1) patients had risk factors for pulmonary aspergillosis (such as neutropenia, transplantation, and immunosuppressive therapy), (2) patients had certain clinical manifestations of IPA, (3) imaging results were abnormal, and (4) there was mycological evidence of IPA [15, 17]. The GM antigen criteria used for probable IPA diagnosis are consistent with the revised EORTC/MSGERC criteria, which require a GM level of ≥ 1.0 optical density (OD) in single serum or plasma, ≥ 1.0 in BAL fluid, or ≥ 0.7 in single serum or plasma and ≥ 0.8 in BAL fluid [15]. *Aspergillus* polymerase chain reaction (PCR) was not used as it was unavailable at the institutes. Both proven and probable IPA cases were included in this study. The lower and higher *Asp*-IgG cut-off levels were set at 40 mgA/L, based on the cut-off level used for chronic pulmonary aspergillosis [14].

For outcome definition, survivor group was defined as those who survived beyond 90 days after IPA diagnosis while non-survivor group was those who died within 90 days after IPA diagnosis. High dose steroid user was defined as those who received at least 20 mg/day equivalent of oral prednisolone.

Two independent chest specialists who were blinded to the *Asp*-IgG results reviewed the clinical records and images of participants and determined the presence of IPA. In case of discrepancies, the specialists discussed and arrived at a consensus.

### Systematic review

The PRISMA flowchart depicted in Supplementary Fig. 1 outlines the process used to search for relevant studies on the use of *Aspergillus* IgG in the diagnosis and prediction of outcomes in invasive pulmonary aspergillosis. A keyword search was conducted on the PubMed and EMBASE databases using the text terms "invasive pulmonary aspergillosis" and "*Aspergillus* IgG".

### Statistical analysis

We used statistical methods to compare differences between groups, including independent-sampled t test or one-way ANOVA for continuous variables with normal distribution and homogeneity of variance, and non-parametric Mann–Whitney U test or Kruskal–Wallis test for other continuous variables. For categorical variables, we used the chi-square test. Primary outcome was overall survival. Secondary outcome was 90-day mortality rate. To compare and analyze overall survival between high and low *Asp*-IgG levels, we used the log-rank test. We used multivariable Cox regression to explore factors associated with overall survival. To explore factors associated with 90-day mortality, we used logistic regression analysis. The multivariable model adjusted for variables such as age, sex, demographic data, serum GM level, and laboratory data and followed stepwise selection with entry and stay criteria set at 0.2 and 0.1, respectively. All statistical analyses were performed using SAS version 9.4 (SAS Institute Inc, Cary, NC, USA).

## Results

### Clinical characteristics of participants in the IPA cohort

During the study period, a total of 40 participants were enrolled in the IPA cohort, and their clinical characteristics are presented in Table 1. Among the IPA patients, diabetes mellitus was the most frequent underlying disease (*n* = 19, 47.5%), followed by cancer and systemic steroid use, each affecting 13 participants (32.5%). While Taiwan had relatively low rates of COVID-19 during the study period, none of the IPA cases were associated with COVID-19-associated pulmonary aspergillosis (CAPA). Instead, 8 patients (20%) were diagnosed with influenza-associated pulmonary aspergillosis (IAPA). Underlying respiratory conditions include chronic obstructive pulmonary disease (COPD) (*n* = 10, 25%), followed by bronchiectasis (*n* = 7, 17.5%), asthma (*n* = 6, 15%) and idiopathic pulmonary fibrosis (*n* = 3, 7.5%). Among the IPA patients, 10 participants (25%) exhibited a hypodense sign and 9 individuals (22.5%) had cavitation/air-crescent sign on their chest CT images.

**Table 1:** Clinical characteristics of 40 participants

	All (*n* = 40)	*Asp*-IgG ≤ 40 mgA/L (*n* = 29)	*Asp*-IgG > 40 mgA/L (*n* = 11)	*p* value	Survival Group (*n* = 14)	Non-Survival Group (*n* = 26)	*p* value
Age	67.0 ± 16.4	70.0 ± 16.1	59.2 ± 15.1	0.061	57.5 ± 18.3	72.2 ± 12.9	0.005
< 45	4 (10)	2 (6.9)	2 (18.2)	0.058	3 (21.4)	1 (3.9)	0.071
45–65	13 (32.5)	7 (24.1)	6 (54.6)		6 (42.9)	7 (26.9)	
> 65	23 (57.5)	20 (79.0)	3 (27.3)		5 (35.7)	18 (69.2)	
Body mass index	22.4 ± 4.62	22.6 ± 3.79	21.8 ± 6.51	0.688	23.5 ± 5.65	21.8 ± 3.97	0.293
< 18	8 (20)	3 (10.3)	5 (45.5)	0.528	2 (14.3)	6 (23.1)	0.487
18–25	22 (55)	19 (65.5)	3 (27.3)		7 (50.0)	15 (57.7)	
> 25	10 (25)	7 (24.1)	3 (27.3)		5 (35.7)	5 (19.2)	
Male	26 (65)	18 (62.1)	8 (72.7)	0.539	6 (42.9)	20 (76.9)	0.312
Smoking	18 (45)	12 (41.4)	6 (54.6)	0.455	4 (28.6)	14 (53.9)	0.125
Underlying conditions							
DM	19 (47.5)	15 (51.7)	4 (20)	0.385	4 (28.6)	15 (57.7)	0.105
Malignancy	13 (32.5)	10 (34.5)	3 (27.3)	0.664	3 (21.4)	10 (38.5)	0.316
Post-influenza	8 (20)	5 (17.2)	3 (27.3)	0.479	3 (21.4)	5 (19.2)	0.868
Autoimmune disease	7 (17.5)	6 (20.7)	1 (9.1)	0.656	1 (7.14)	6 (23.1)	0.387
ESRD	4 (10)	4 (13.8)	0	0.194	1 (7.14)	3 (11.5)	0.659
COPD	10 (25)	8 (27.6)	2 (18.2)	0.696	2 (14.3)	8 (30.8)	0.446
Asthma	6 (15)	6 (20.7)	0	0.162	3 (21.4)	3 (11.5)	0.646
Bronchiectasis	7 (17.5)	4 (13.8)	3 (27.3)	0.369	3 (21.4)	4 (15.4)	0.679
Idiopathic pulmonary fibrosis	3 (7.5)	3 (10.3)	0	0.548	1 (7.1)	2 (7.7)	1.0000
Systemic steroids	13 (32.5)	11 (37.9)	2 (18.2)	0.234	1 (7.14)	12 (46.2)	0.015
High dose steroid user	5 (12.5)	4 (13.8)	1 (9.1)	1.000	1 (7.1)	4 (15.4)	0.640
Immunosuppressant*	10 (25)	8 (27.6)	2 (18.2)	0.540	2 (14.3)	8 (30.8)	0.251
Inhaled steroids	7 (17.5)	5 (17.2)	2 (18.2)	0.944	3 (21.4)	4 (15.4)	0.631
Definite IPA	10 (25)	6 (20.7)	4 (36.4)	0.307	3 (21.4)	7 (26.9)	0.702
Probable IPA	30 (75)	23 (79.3)	7 (63.6)	0.307	11 (78.6)	19 (73.1)	0.702
*Aspergillus* culture positivity	16 (40)	9 (31.0)	7 (63.6)	0.060	7 (50)	9 (34.6)	0.344
Serum GM (Index)	1.49 ± 1.74	1.21 ± 1.51	2.22 ± 2.13	0.101	1.47 ± 1.90	1.50 ± 1.68	0.960
BAL GM (Index)	4.50 ± 7.75	2.52 ± 2.02	8.9 ± 13.0	0.180	7.61 ± 12.1	2.60 ± 1.85	0.200
Chest image findings							
Hypodense sign	10 (25)	6 (20.7)	4 (36.4)	0.307	3 (21.4)	7 (26.9)	0.702
Cavitation/Air crescent sign	9 (22.5)	6 (20.7)	3 (27.3)	0.656	4 (28.6)	5 (19.2)	0.500
Consolidation	34 (85)	24 (87.8)	10 (90.9)	0.519	11 (78.6)	23 (88.5)	0.403
Ground-glass opacity	17 (42.5)	11 (37.9)	6 (54.6)	0.343	8 (57.1)	9 (34.6)	0.169
*Aspergillus* fumigatus IgG titer (mgA/L)	33.5 ± 47.2	12.5 ± 9.47	89.0 ± 61.4	0.002	41.8 ± 53.8	29.0 ± 43.7	0.420
*Aspergillus* niger IgG titer (mgA/L)	19.7 ± 22.5	9.16 ± 7.76	46.7 ± 25.5	0.001	21.5 ± 21.5	18.8 ± 23.4	0.718
Laboratory data							
WBC (× 10^3^/µL)	11.1 (7.4–16.6)	11.3 (8.45–16.7)	9.23 (5.78–13.9)	0.250	6.48 (5.68–13.9)	12.3 (9.71–17.6)	0.007
ANC (× 10^3^/µL)	6.26 (2.84–11.1)	5.33 (2.14–9.66)	7.75 (4.40–12.8)	0.256	4.51 (4.25–6.84)	7.97 (2.14–11.7)	0.571
Hemoglobin (g/dL)	9.3 (8.3–11.4)	9.1 (8.2–11.4)	9.7 (8.4–11.3)	0.682	10.2 (8.2–13.1)	8.6 (8.4–10.4)	0.152
Platelet (× 10^3^/μL)	175 (84.5–278.5)	122 (83–217)	275 (134–339)	0.033	202.5 (134–325)	113 (83–255)	0.092
Albumin (g/dL)	3.2 (2.65–3.8)	3.5 (3.9–2.7)	2.9 (2.6–3.3)	0.163	3.6 (3.1–4.0)	2.9 (2.5–3.6)	0.016
C-reactive Protein (mg/dL)	6.85 (3.70–13.1)	6.64 (4.48–11.1)	9.86 (1.90–24.3)	0.477	4.05 (1.85–9.86)	7.59 (4.76–13.2)	0.281

*ANC* Absolute neutrophil count, *BAL* Bronchoalveolar lavage, *CT* Computed tomography, *COPD* Chronic obstructive pulmonary disease, *DM* Diabetes mellitus, *ESRD* End-stage renal disease, *GM* Galactomannan, *IPA* Invasive pulmonary aspergillosis, *TB* Tuberculosis, *WBC* White blood cell

Data are either mean ± standard deviation or number (percentage). For laboratory data, data are expressed as median (interquartile range, Q1-Q3)

^*^ Immunosuppressants includes anti-cancer chemotherapy agents, disease-modifying antirheumatic drugs (DMARDs) and anti-rejection drugs

In the IPA cohort, 16 (40%) patients had high serum GM level (OD > 1) while 24 (60%) patients had low serum GM level (OD < 1). Eleven patients (27.5%) had high *Asp*-IgG levels (> 40 mgA/L) while 29 patients (72.5%) had low *Asp*-IgG levels. Patients with high *Asp*-IgG levels had a higher platelet count compared to those with low levels. Out of the total cohort, 14 patients (35%) survived at the 90th day, while 26 patients (65%) did not. The non-survivors were typically older, more likely to have received systemic steroids, and had higher white blood cell counts but lower albumin levels than the survivors.

### Asp-IgG and IPA outcome

**Fig. 2:**
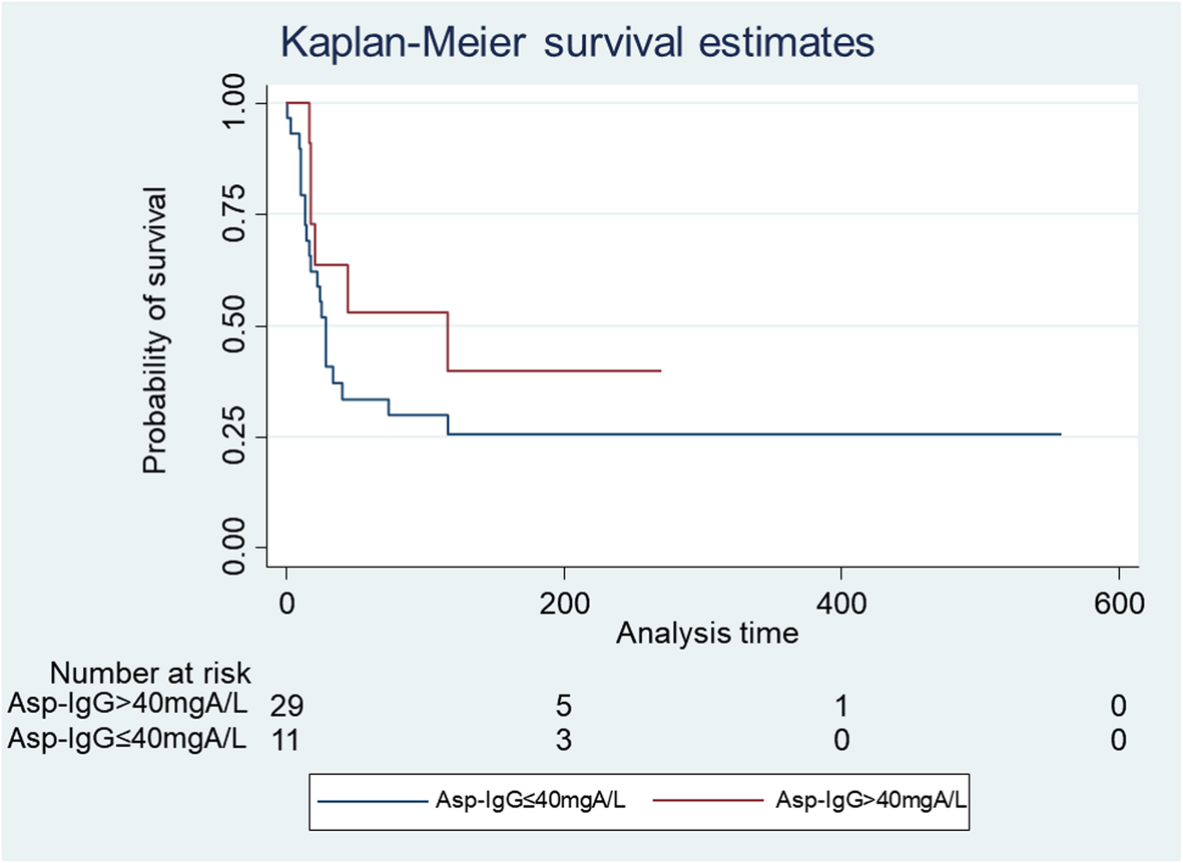
Kaplan–Meier curve of high and low Asp-IgG level

In the log-rank test, high *Asp*-IgG levels were borderline associated with improved overall survival (HR: 0.41, 95% CI: 0.11–1.54, *p* = 0.173). Figure 2 illustrates the Kaplan–Meier curve of participants with high and low Asp-IgG level. Higher *Asp*-IgG levels were associated with better overall survival (HR: 0.22, 95% CI: 0.05–0.99, *p* = 0.035) in multivariable Cox regression analysis after adjusting for confounding factors.

Although not statistically significant, there was a trend towards lower 90-day mortality (45.5% vs. 72.4%, OR: 0.32, 95% CI: 0.08–1.34, *p* = 0.118) in patients with high *Asp*-IgG. Only age was found to be significantly associated with higher risk for 90-day mortality (OR: 1.07, 95% CI: 1.01–1.12, *p* = 0.015) in multivariable logistic regression analysis.

### Asp-IgG titer association and correlation with different level of serum GM, BAL GM and Aspergillus culture-positivity

**Fig. 3:**
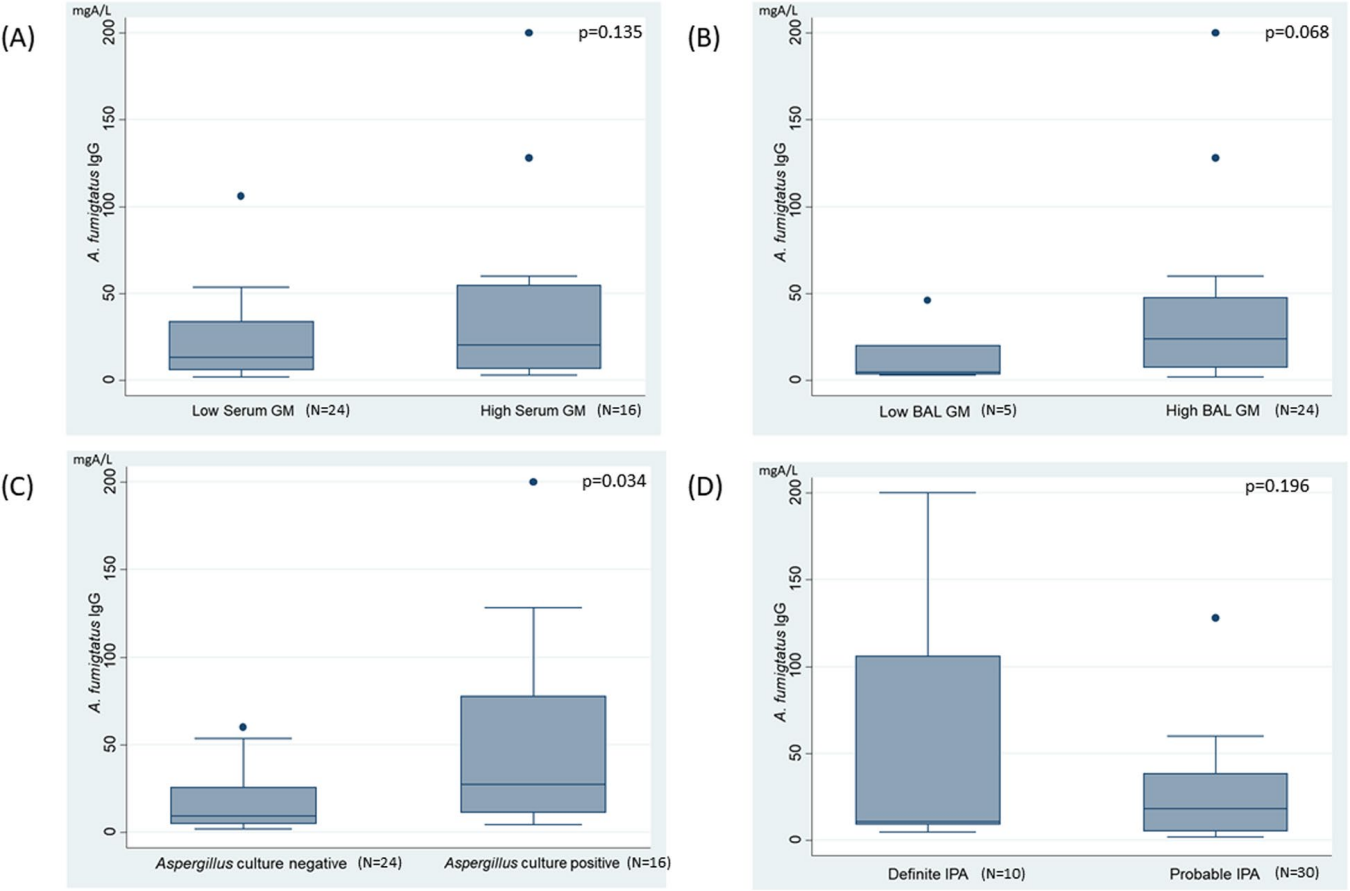
Aspergillus-specific IgG level in patients with different level of serum GM (**A**), BAL GM (**B**), *Aspergillus* culture-positivity (**C**) and disease entity (**D**)

The titers of *Asp*-IgG among different levels of serum GM, BAL GM, Aspergillus culture-positivity and disease entity are illustrated in the Fig. 3. We observed that the *Asp*-IgG titers were higher in patients with positive *Aspergillus* culture than in those with negative culture results (56.9 ± 65.9 vs 17.9 ± 17.7 mgA/L, *p* = 0.034). Patients with high serum GM levels also tended to have higher *Asp*-IgG titers than those with low serum GM levels, although the difference was not statistically significant (49.8 ± 66.7 vs 22.6 ± 23.8 mgA/L, *p* = 0.135). Patients with high BAL GM levels also showed a trend toward higher *Asp*-IgG titers compared to those with low BAL GM levels (42.5 ± 55.7 vs 15.4 ± 18.5 mgA/L, *p* = 0.068).

### IPA outcome stratified according to serum GM and Asp-IgG

When stratified by GM serum levels and *Asp*-IgG titers, the highest 90-day mortality rate was observed in the group with low *Asp*-IgG levels (≤ 40 mgA/L) and high serum GM levels (OD > 1) (80%, 8/10), followed by the group with low *Asp*-IgG levels and low serum GM levels (68.4%, 13/21). The lowest 90-day mortality rate was observed in the group with high *Asp*-IgG levels and low serum GM levels (40%, 2/5).

### Asp-IgG among IPA patients with different respiratory diseases

The titers of Asp-IgG were 53.5 ± 67.3 mgA/L in patients with bronchiectasis (*n* = 7), 41.4 ± 39.2 mgA/L in patients recovered from influenza (*n* = 8), 30.2 ± 30.8 mgA/L in those with COPD (*n* = 10), 12.8 ± 6.47 mgA/L in those with idiopathic pulmonary fibrosis (*n* = 3) and 12.6 ± 10.1 mgA/L in those with asthma (*n* = 6).

### Factors associated with Asp-IgG positivity in IPA cohort

The results of the multivariable logistic regression analysis showed that a higher platelet count (OR: 1.01 for every 1 × 10^3^/μL increase, 95% CI: 1.003–1.016, *p* = 0.043) was associated with an increased risk of *Asp*-IgG positivity in the IPA cohort. Additionally, there was a borderline association between *Aspergillus* culture positivity and an increased risk of *Asp*-IgG positivity (OR: 4.57, 95% CI: 0.93–22.5, *p* = 0.062).

### Meta-analysis results

The summarized results of published studies regarding Asp-IgG among IPA patients in meta-analysis is described in Table 2. The meta-analysis included 6 studies, with 2 using Dynamiker and 2 using ImmunoCap *Asp*-IgG kits. Although quantitative meta-analysis was not possible due to variations in cut-off levels and different *Asp*-IgG kits, studies consistently reported a high prevalence of *Asp*-IgG (45–80%) in non-neutropenic IPA patients using pre-specified cut-off points with commercial kits. In these studies, the sensitivity of serum GM antigen (19–46%) for IPA diagnosis was lower than that of *Asp*-IgG. Regarding *Asp*-IgG and IPA outcome, only one study has compared between IPA survivors and non-survivors and found no significance difference albeit that *Asp*-IgG was slightly higher in the survival group (*p* = 0.268) [12].

**Table 2:** Summarized results of published studies regarding Asp-IgG among IPA patients

	Patient population	Manufacturer of kit	Cut-off point of serum GM	Sensitivity of serum GM	Cut-off level of IgG used/proposed	Seroprevalence of *Asp*-IgG in IPA
Tirelli C et al. 2022 [32]	IPA based on positive BAL *Aspergillus* culture (*n* = 97)	ImmunoCap	0.5	NA	22.6 mgA/L	74%
	IPA based on positive serum GM (*n* = 39)		0.5	NA	23.8 mgA/L	78%
Yu Q et al. 2020 [11]	IPA (*n* = 37)	Dynamiker	0.85*	29.7%	134.46 AU/ml	45.9%
Dobias R et al. 2018 [12]	Proven IPA (*n* = 10), Putative IPA (*n* = 31) and non-IPA colonization (*n* = 46)	Immunolab	0.5	46.3%	1.2 positivity index value	80.5%
Lu et al. [33]	IPA (*n* = 99)	Dynamiker	1	19.2%	80 AU/ml	59.6%
Hsiao et al. [34]	IPA (*n* = 6)	ImmunoCap	0.5	33.3%	9.73 mgA/L	66.7%
Jiang et al. [35]	CAPA (*n* = 8)	In-house ELISA	0.5	87.5%	1 index	100%
This study	IPA (*n* = 40)	ImmunoCap	1 0.5	40% 52.5%	40	27.5%

AUC, area under curve; CAP, community-acquired pneumonia; CAPA, COVID-19 associated pulmonary aspergillosis; ELISA, Enzyme-linked immunosorbent assay; IPA, invasive pulmonary aspergillosis

^*^GM test performed with commercial enzyme-linked immunosorbentassay (ELISA) kit (Dynamiker Biotechnology Co., Ltd

Tianjin, China); otherwise all other GM test was performed with Bio-Rad Platelia kit

## Discussion

Our study found that *Asp*-IgG was positive in about one fourth of IPA patients in a population without hematologic malignancy and neutropenia. The sensitivity of *Asp*-IgG (27.5%) was lower than that of the serum GM antigen (40%) with an OD greater than 1. This study also revealed that higher *Asp*-IgG was associated with better survival, and the worst outcome was observed among those with positive serum GM and low *Asp*-IgG. Additionally, the *Asp*-IgG titer was higher among IPA patients with *Aspergillus* culture positivity.

Aside from diagnosis, *Asp*-IgG has been proposed to be a useful monitoring biomarker for treatment response in CPA [18]. In fungus studies, fungal-specific antibodies could pose direct protective actions by acting on fungal cells and through phagocytosis and complement activation [19]. In another study, *Aspergillus* IgA was higher among IPA patients with survival compared with non-survival patients whereas *Asp*-IgG level was higher in the survival group numerically but without statistical significance achieved [12].

There were some possible explanations for the association between better higher *Asp*-IgG with higher survival. The production of *Asp*-IgG may indicate recovery from acute stage. In our study, patients with high serum GM and low *Asp*-IgG had the highest 90-day mortality rate while those with low serum GM and high *Asp*-IgG had the lowest 90-day mortality rate. While serum GM may indicate fungal burden and disease severity, the level of *Asp*-IgG may indicate the degree of immune reaction to disease. Interestingly, in animal study, *Asp*-IgG level was higher among those who received two doses of *Aspergillus* than those with single high dose or control. Whether the higher *Asp*-IgG level represents previous exposure to Aspergillus with remained immunity is worth investigation [20]. Additionally, older patients were known to have reduced antibody production to vaccination and diseases [21]. Though we have adjusted age in our final model, whether *Asp*-IgG is an intermediate marker reflecting better general condition which then contributed to improved survival remained unknown.

Platelet count was associated with IgG positivity among IPA patients. While in infectious diseases, increased platelet count has been reported in the convalescent phase [22]. The association between increased platelet count and IgG positivity among IPA patients may indicate recovery from the acute stage. Additionally, platelet is involved in immunity and has been playing a protective role against aspergillosis. Platelet can be activated by *Aspergillus* and in turn damage *Aspergillus* hyphae [23, 24]. While the difference of platelet count between groups may be relatively subtle from clinical point of view, the potential association between higher platelet count, higher *Asp*-IgG and better outcome warrants further investigation.

*Aspergillus* culture positivity was also associated with higher *Asp*-IgG level. The existence of *Aspergillus* may suggest chronic exposure to *Aspergillus* and possible chronic colonization and burden of *Aspergillus* in respiratory tract. IPA may be secondary to the acute flare-up or exacerbation of existing *Aspergillus* in human respiratory tracts, along with the finding of elevated *Asp*-IgG in this group of patients [25]. Further supporting this theory is the observation that, despite the relatively small sample size, patients with underlying structural lung diseases such as bronchiectasis exhibited higher *Asp*-IgG levels compared to patients with other respiratory diseases in our study. *Aspergillus* may colonize the airways of patients with structural lung diseases, potentially leading to elevated *Asp*-IgG levels in this particular disease category [25].

Serum GM was considered to be an important diagnostic test for IPA. Notably, the performance of serum GM may be limited in non-neutropenic patients since the sensitivity of GM in this group was reduced [26, 27]. In one study at a cut-off level of OD value of 0.5, the sensitivity of serum GM was only 37.8% [27]. While EORTC/MSGERC criteria has heightened the serum GM cut-off level to 1 OD this may cause further reduction of sensitivity by GM in the non-neutropenic population. Non-neutropenic hosts, however, are now recognized as an increasing important population, especially in the ICU setting [28]. Our study, therefore, could help aid in currently limited diagnostic tests for IPA among non-neutropenic hosts.

There were no severe acute respiratory syndrome coronavirus 2 infection (COVID-19)-associated pulmonary aspergillosis (CAPA) patients in our studies. On the other hand, there were one fifth patients with IAPA in our IPA cohort. While CAPA was considered to be an emerging disease during the COVID-19 pandemic, Taiwan was relatively free from COVID-19 during our study period due to rapid response, intact public health system infrastructure and strict infection control measures [29]. This leads to the paucity of CAPA patients in our study and we are therefore unable to analyze *Asp-*IgG among CAPA patients. The degree of viral lung damage and underlying immunosuppression (dose and duration of steroid or immunosuppressant), however, varies in CAPA, making it difficult to analyze the true risk factors of aspergillosis [30]. CAPA, therefore, may be at best discussed individually.

The performance of *Asp*-IgG in IPA has been tested in previous studies [11, 12]. Indeed, *Asp*-IgG may not be comparable to serum or BAL GM antigen test, especially given the fact that GM test is an essential part of IPA diagnosis [15]. Furthermore, previous studies regarding *Asp*-IgG in IPA were mainly conducted in non-neutropenic patients because antibody production was considered to be defective in neutropenic patients [11, 12]. In a more recent study, using a cut-off level of 22.6 mgA/L for *Asp-*IgG, the seropositive rate was 74% among *Aspergillus* BAL culture positive IPA patients while the AUC for IPA diagnosis remained modest (AUC: 0.62). In the study by Dobias et al., the involvement of *Asp-*IgG and IgA test assay could improve IPA diagnosis in non‐neutropenic patients by increasing the sensitivity and negative predictive value when combined with the GM or (1,3)‐β‐d‐glucan assays [12].

Our study also has limitations. First, this study was conducted in Taiwanese population with limited sample size and cross-ethnicity validation was not readily available. Our study, however, may be exploratory in nature and we believe that our study could point out knowledge gaps for future studies to fill. Second, we did not have *Aspergillus* PCR in our facilities. This may lead to the under-estimation of IPA while the revised EORTC criteria endorsed the use of PCR study [15]. Third, this *Aspergillus*-specific IgG was performed using ImmunoCap kit. While there were many other commercial kits in the market, whether our findings could be applicable to other commercial *Aspergillus* IgG kits remained unknown [31].

In conclusion, the sensitivity of serum *Aspergillus*-specific IgG may be lower than serum GM in non-neutropenic IPA patients. *Aspergillus*-specific IgG may also be used as a prognostic tool and indicator of disease outcome while future large-scale studies were warranted to validate our findings.

## Supplementary Information

Supplementary Material 1.

## Data Availability

All data were available for sharing upon reasonable request.

## Abbreviations

BAL: bronchoalveolar lavage
CAPA: severe acute respiratory syndrome coronavirus 2 infection-associated pulmonary aspergillosis\
COPD: chronic obstructive pulmonary disease
COVID: Coronavirus disease
EORTC/MSGERC: European Organization for Research and Treatment of Cancer and the Mycoses Study Group Education and Research Consortium
GM: galactomannan
IAPA: influenza-associated pulmonary aspergillosis
ICU: intensive care unit
IPA: invasive pulmonary aspergillosis
OD: optical density
PCR: polymerase chain reaction
