# Supplementary material for: Seroprevalence and prognostic value of Aspergillus-specific IgG among non-neutropenic invasive pulmonary aspergillosis patients: a prospective multicenter study
Source: Pneumonia (Nathan). 2024 Nov 5;16:28. doi: 10.1186/s41479-024-00154-8 (PMC11536880; doi:10.1186/s41479-024-00154-8)
Supplement: Supplementary file 1 — Supplementary Material 1. [file 41479_2024_154_MOESM1_ESM.docx]

**Supplementary Figure 1.** PRISMA flowchart of systematic review

**Identification of studies via databases and registers**

Records identified from

Pubmed (n = 52)

EMBASE (n = 12)

**Identification**

Duplicate records removed (n = 6)

Records screened

(n = 58)

Reports sought for retrieval

(n = 58)

**Screening**

Reports excluded:

Case report (n=7)

Review article (n=10)

Did not involve humans (n=6)

Only chronic pulmonary aspergillosis included (n=25)

*Asp*-IgG not checked (n=3)

*Asp*-IgG method not specified (n=1)

Reports assessed for eligibility

(n = 58)

Studies included in review

(n = 6)

**Included**
